# Military Notes

**Published:** 1918-02

**Authors:** 


**A Monthly Review of Medicine and Surgery**

EDITOR AND PUBLISHER, DR. A. L. BENEDICT, 228 Summer St., corner of Elmwood Ave., Buffalo, (Address for all communications. Please make personal and telephone calls before 1 P. M.)

Third, in age, on the western continent. Independent, but supports professional organizations. News limited mainly to Western N. Y. and Penn.

Subscription $2.00 a year; $3.00 for two years in advance. Changes and errors in address or failure or duplication of delivery, should be reported immediately.

Yearly Volume 73 FEBRUARY, 1918 Number 7

### Military Notes.

War bread has arrived. For some time, the bread making has been simplified to a basis of plain wheat flour, a very little salt and about half the quantity of yeast used in domestic cooking and very much less "raising" than that of ordinary bakers whose aim is to produce a bread that is good when perfectly fresh but that will encourage business by becoming stale in a few hours. The appliances have also been simplified to the minimum available at the front and part of the baking has been done in clay ovens which could be extemporized anywhere, though, as a matter of convenience, most of the baking has been done in more permanent ovens. The composition of the war bread is a matter of dispute. It is, if possible, even more palatable than the white bread which is still alternated with it. So far as can be judged by the taste, it is a whole wheat bread but some claim that it contains some other cereal, corn or oats or both.

Complaints as to army rations seem to be inversely proportionate to the scale of living of the individual at home. The palatability of different masses varies considerably but, except for minor points of difference, the messes for enlisted men at a cost of a trifle less than 40 cents a day are equal to those of officers who pay from 75 cents to a dollar. The higher-priced messes, however, usually include a liberal provision for hospitality without extra charge and various incidental expenses. Some messes even have paper napkins and there are rumors of table linen at a few. Privates from wealthy families usually speak well of their messes. Those from humble surroundings, often express the feeling that they are very inadequately nourished. Mess sergeants declare that patients eat more heartily than men on active duty and a surprisingly few patients in military hospitals require any particular selection of diet, or show any loss of appetite. The war bread was almost unanimously regarded as excellent and the consumption on the first day of its appearance was enormous.

The avoidance of waste is reasonably thorough though certain camps are compelled to destroy much theoretically valuable waste in the form of manure, tin cans, etc., on account of lack of local demand and prohibitive freight rates to points at which a commercial demand exists. Most of the real waste is due to personal carelessness which no system of control can cover perfectly. Combustible waste of all kinds is used for heating, though newspapers may be utilized indirectly for lining walls or even clothing and thus conserving calories. As an illustration of the possible limitation of waste, may be mentioned a mess feeding about 200 men, at which the actual waste of bread—plate leavings which could not be saved for dressings, puddings, etc.—weighed less than 2 pounds for an entire day.

A comparison for periods of a week, showed a difference between an attempt to conserve sugar to a degree established by regulations, and its unlimited use, of about 1/25 pound per capita per diem.

Mental defectives, not detected by initial examinations of recruits but sorted out by expert boards from men who have shown marked aberration or who have failed to make good in several months of military training, comprise a trifle less than 1% of the total number of average troops. They include a considerable number of cases of late tertiary syphilis of the central nervous system, a few cases of genuine insanity of various types, some cases of hysteria mostly involving subconscious malingering but the great majority of this fraction of a per cent. are cases of imperfect mental development with which modern psychiatry is especially concerned.

Tuberculosis has come to have almost a special military definition. Very rarely do well developed cases pass the initial examination nor, in spite of exposure, the frequent development of severe colds and a moderate though very variable incidence of pneumonia, does tuberculosis develop in troops in cantonments in this country, in such types as are common in civil practice, either medical or surgical. Even incipient tuberculosis with marked anaemia and general weakness, is rather uncommon. From the military standpoint, with due regard to the expected stress of trench fighting and the opportunities for infection in a terrain in which European armies with a rather high incidence of tuberculosis have operated in large numbers for three years, it has been deemed wise to weed out all with any pulmonary secretion that can be detected by the method of inspiration, complete expiration and cough. The impossibility of securing quiet for examination of large numbers of men, renders percussion of comparatively little value, although practiced rather as a method of palpation than audition. Tuberculin tests of various kinds are not employed, partly because of obvious technical difficulties, partly because of the question recently raised as to whether such reactions are genuinely specific or due to susceptibility to foreign proteins in general. Direct bacteriologic examinations are of course provided for in laboratories but these deal with a minority of cases which represent a singularly unfortunate and rare development of a more or less pardonable error on the part of examining boards. In general, there is no sputum available for examination. Thus, the cases designated as tuberculosis include many suspects and comparatively few which will not recover under favorable hygienic conditions. Pending the inevitable delay in securing their discharge from the army—granting that they are not soon restored to health—they have the advantage of abundant food, support, medical, if not necessarily medicinal, treatment and training as to precautions in their own interests and to prevent the spread of infection.

